# Severe thrombocytopenia following exposure to orlistat, alkylphenol, and pyrethroids: coincidence or causation?

**DOI:** 10.17843/rpmesp.2025.423.14626

**Published:** 2025-08-13

**Authors:** Anais Zapata-Rafael, Johan Azañero-Haro, Alonso Soto

**Affiliations:** 1 Departamento de Medicina Interna. Hospital Nacional Hipólito Unánue, Lima, Peru. Departamento de Medicina Interna Hospital Nacional Hipólito Unánue Lima Peru; 2 Universidad Científica del Sur, Lima, Peru. Universidad Científica del Sur Universidad Científica del Sur Lima Peru

Mr. Editor. Severe thrombocytopenia, defined as a platelet count below 20,000/μL, is a life-threatening hematological condition that can arise from immunological, infectious, neoplastic, pharmacological, or toxic causes. In this context, we present the case of a patient recently exposed to substances with hematotoxic potential. The patient’s clinical evolution raises relevant diagnostic questions and could contribute to the recognition of unusual triggers of severe thrombocytopenia.

The patient is a 32-year-old farmer who lives in El Agustino, an urban district of Metropolitan Lima. Due to his occupation, he is chronically exposed to agrochemicals. He came to the emergency department with spontaneous gingivorrhagia, petechiae, and multiple ecchymoses. On admission, his platelet count was undetectable (0/μL), with no evidence of active infection or other significant hematological alterations.

Among the relevant history, the patient reported the recent use of orlistat for seven consecutive days (120 mg three times a day), without medical indication, for the purpose of losing weight. He had grade I obesity, with a body mass index (BMI) of 32.6 kg/m² (Weight: 92 kg; height: 1.68 m). He reported no liver or metabolic diseases, nor relevant comorbidities. Additionally, three days before the onset of symptoms, he had manually sprayed his crops with the agrochemicals: SuperWet® (Alkylphenol ethoxylate) and Delmur® (Pyrethroid), for approximately 4 hours daily and without using personal protective equipment. The exposure included direct skin contact (hands, neck, face) and the respiratory route, without a mask or waterproof clothing, which caused a sensation of nasal irritation and exposure to intense odors.

He denied any personal or family history of autoimmune or hematological diseases. He was hospitalized for seven days. Treatment was initiated with intravenous methylprednisolone 1g/day for four days, followed by oral prednisone at 1 mg/kg/day in a tapering schedule. Simultaneously, orlistat was discontinued, and exposure to chemicals was stopped. At 72 hours, he showed a rapid increase in platelet count to 65,000/μL, with resolution of symptoms. On the fifth day, his platelets reached 100,000/μL, with no evidence of active bleeding, so it was decided to discharge him with oral treatment and outpatient follow-up.

Regarding complementary studies, infectious and hemolytic causes were ruled out. The results showed negative ANA, negative direct and indirect Coombs test, LDH of 170 U/L (reference value <185), and bilirubin levels within normal ranges. Haptoglobin and antiplatelet antibodies were not available due to laboratory limitations. A bone marrow study was not performed either, given the early rise in the platelet count and the favorable clinical response. During the outpatient follow-up, which was achieved until day 14 post-discharge, no recurrence was evident: the platelet count was 121,000/μL on day 7 and 157,000/μL on day 14 after discharge. Subsequently, the patient did not return for check-ups and was lost to follow-up.

Although the clinical presentation was compatible with autoimmune thrombocytopenic purpura (ATP), the absence of a history of autoimmune diseases and the recent exposure to drugs and agrochemicals compel the consideration of a broader spectrum of etiologies. The recovery from thrombocytopenia coincided temporally with both the administration of corticosteroids and the discontinuation of orlistat and agrochemicals, which makes it impossible to establish with certainty whether the remission was the result of the immunosuppressive treatment, the withdrawal of toxic agents, or a combination of both factors. This diagnostic ambiguity is the focus of this communication, aimed at fostering academic discussion on potential, poorly described immuno-toxic mechanisms.

While orlistat—a pancreatic lipase inhibitor used in obesity management—has been scarcely linked to thrombocytopenia in the literature, some reports suggest a possible association. One of the few described cases involves a woman with grade III obesity who developed reversible thrombocytopenia after discontinuing the drug ^1^. However, the pathophysiological mechanism of this relationship is not yet clearly defined. It has been proposed that orlistat could induce the formation of drug-dependent antibodies that react with platelet glycoproteins such as GP Ib/IX and GP IIb/IIIa, promoting platelet destruction. Some authors also suggest that certain medications could interfere with platelet production by altering megakaryocyte function ^2^.

On the other hand, the relationship between alkylphenols and hematological alterations in humans is not well-established ^3^. However, studies in animal models have shown that these substances can induce systemic inflammation and multi-organ dysfunction, which could indirectly contribute to hematological alterations. In the case of pyrethroids such as deltamethrin, the described toxicity has been primarily neurological, with manifestations such as choreoathetosis, excessive salivation, tremors, and motor symptoms ^4^. Nevertheless, isolated cases of mild thrombocytopenia have been reported in situations of severe poisoning ^5^.

The simultaneous exposure to orlistat, alkylphenols, and pyrethroids adds further complexity to this case. Although there is no direct evidence to suggest a synergistic effect of these substances on hematopoiesis, it cannot be ruled out that, together, they may act as triggers in genetically predisposed individuals. The absence of acute infections and other identifiable causes to explain the severe thrombocytopenia reinforces the need to consider these substances within the differential diagnosis, especially in contexts where self-medication or environmental exposure to pesticides is common.

The presented case highlights the importance of a multidisciplinary approach in managing severe hematological diseases of uncertain etiology, as well as the importance of an exhaustive clinical history. In particular, it underscores the need to consider pharmacological and toxic factors as possible causes of severe thrombocytopenia, especially in patients with a history of self-medication or environmental or occupational exposures, as occurred in our patient. While corticosteroids are effective in the treatment of ATP, a reasonable doubt remains as to whether the recovery of the platelet count was exclusively attributable to the discontinuation of the involved agents or if corticosteroids played a decisive role by inducing the reversibility of the condition.

In this regard, this communication aims to draw attention to the possible involvement of understudied substances in the genesis of severe thrombocytopenia and to stimulate the systematic documentation of similar cases. Finally, the worrying availability of drugs such as orlistat without a medical prescription and the indiscriminate use of pesticides without adequate protective measures should draw the attention of health authorities and regulatory bodies to prevent adverse events of an avoidable origin.
