# Supplementary material for: Severe thrombocytopenia following exposure to orlistat, alkylphenol, and pyrethroids: coincidence or causation?
Source: Rev Peru Med Exp Salud Publica. 2025 Aug 13;42(3):331–2. doi: 10.17843/rpmesp.2025.423.14626 (PMC12679972; doi:10.17843/rpmesp.2025.423.14626)
Supplement: Translation

# Translation: Plaquetopenia severa tras exposición a orlistat, alquilfenol y piretroides: ¿casualidad o causalidad?

**Authors:** Anais Zapata-Rafael, Johan Azañero-Haro, Alonso Soto

**Affiliations:** 1 Department of Internal Medicine. Hospital Nacional Hipólito Unánue, Lima, Perú.; 2 Universidad Científica del Sur, Lima, Perú.

Sr. Editor. La plaquetopenia severa, definida como un recuento plaquetario inferior a 20 000/μL, es una condición hematológica potencialmente mortal, que puede originarse por causas inmunológicas, infecciosas, neoplásicas, farmacológica o tóxicas. En este contexto, presentamos el caso de un paciente expuesto recientemente a sustancias con potencial hematotóxico, cuya evolución clínica plantea interrogantes diagnósticos relevantes y podría contribuir al reconocimiento de factores desencadenantes poco habituales de plaquetopenia severa.

Se trata de un agricultor de 32 años, residente en El Agustino, distrito urbano de Lima Metropolitana, quien por su ocupación mantiene exposición crónica a productos agroquímicos. Acudió al servicio de emergencia por presentar gingivorragia espontánea, petequias y múltiples equimosis. Al ingreso, el recuento plaquetario fue indetectable (0/μL), sin evidencia de infección activa ni otras alteraciones hematológicas significativas.

Entre los antecedentes relevantes, el paciente refirió el uso reciente de orlistat durante 7 días consecutivos (120 mg tres veces al día), sin indicación médica, con el objetivo de bajar de peso. Presentaba obesidad grado I, con un índice de masa corporal (IMC) de 32,6 kg/m² (Peso: 92 kg; talla: 1,68 m). No reportaba enfermedades hepáticas, metabólicas ni comorbilidades relevantes. Asimismo, tres días antes del inicio de síntomas, había realizado fumigación manual de sus cultivos con los productos agroquímicos: SuperWet® (Alquilfenol etoxilado) y Delmur® (Piretroide), durante aproximadamente 4 horas diarias y sin utilizar equipos de protección personal. La exposición incluyó contacto cutáneo directo (manos, cuello, rostro) y vía respiratoria, sin mascarilla ni indumentaria impermeable, lo que generó sensación de irritación nasal y exposición a olores intensos.

Negó antecedentes personales o familiares de enfermedades autoinmunes o hematológicas. Fue hospitalizado durante siete días. Se inició tratamiento con metilprednisolona intravenosa 1g/día durante cuatro días, seguido de prednisona oral a 1 mg/kg/día en esquema descendente. Simultáneamente, se suspendió el orlistat y se interrumpió la exposición a químicos. A las 72 horas presentó un rápido incremento del recuento plaquetario a 65 000/μL, con resolución de síntomas. Al quinto día, las plaquetas alcanzaron los 100 000/μL, sin evidencia de sangrado activo, por lo que se decidió su alta con tratamiento oral y seguimiento ambulatorio.

En cuanto a los estudios complementarios, se descartaron causas infecciosas y hemolíticas. Los resultados mostraron ANA negativo, prueba de Coombs directa e indirecta negativas, LDH de 170 U/L (valor de referencia <185) y bilirrubinas dentro de rangos normales. No se contó con haptoglobina ni anticuerpos antiplaquetarios por limitaciones del laboratorio. Tampoco se realizó un estudio de medula ósea, dado el ascenso precoz del recuento plaquetario y la respuesta clínica favorable. Durante el seguimiento ambulatorio, que se logró hasta el día 14 posalta, no se evidenció recurrencia: el recuento plaquetario fue de 121 000/μL al día 7 y 157 000/μL al día 14 después del alta. Posteriormente, el paciente no retornó a los controles y se perdió del seguimiento.

Aunque el cuadro clínico fue compatible con púrpura trombocitopénica autoinmune (PTA), la ausencia de antecedentes de enfermedades autoinmunes y la exposición reciente a fármacos y agroquímicos obliga a considerar un espectro más amplio de etiologías. La recuperación de la plaquetopenia coincidió temporalmente tanto con la administración de corticoides como con la suspensión del orlistat y de los agroquímicos, lo que impide establecer con certeza si la emisión fue resultado del tratamiento inmunosupresor, del retiro de agentes tóxicos, o de ambos factores combinados. Esta ambigüedad diagnóstica constituye el eje de la presente comunicación, orientada a fomentar la discusión académica sobre potenciales mecanismos inmuno-tóxicos poco descritos.

Si bien el orlistat -un inhibidor de la lipasa pancreática utilizado en el manejo de la obesidad- ha sido escasamente vinculado a trombocitopenia en la literatura, existen algunos reportes que sugieren una posible asociación. Uno de los pocos casos descritos corresponde a una mujer con obesidad grado III que desarrolló plaquetopenia reversible tras la suspensión del fármaco ^1^. Sin embargo, el mecanismo fisiopatológico de esta relación aún no está claramente definido, se ha planteado que el orlistat podría inducir la formación de anticuerpos dependientes del fármaco, que reaccionan con glicoproteínas plaquetarias como GP Ib/IX y GP IIb/IIIa, promoviendo la destrucción plaquetaria. Algunos autores también sugieren que determinados medicamentos podrían interferir con la producción plaquetaria mediante la alteración de la función de los megacariocitos ^2^.

Por otro lado, la relación entre los alquifenoles y alteraciones hematológicas en humanos no está bien establecida ^3^. Sin embargo, estudios en modelos animales han mostrado que estas sustancias pueden inducir inflamación sistémica y disfunción múltiorgánica, lo que podría, de forma indirecta, contribuir a las alteraciones hematológicas. En el caso de los piretroides como la deltametrina, la toxicidad descrita ha sido principalmente neurológica, como manifestaciones como coreoatetosis, salivación excesiva, temblores y síntomas motoras ^4^. No obstante, se han reportado casos aislados de plaquetopenia leve en situaciones de intoxicación severa ^5^.

La exposición simultánea a orlistat, alquifenoles y piretroides añade una complejidad adicional al presente caso. Aunque no se encuentra evidencia directa que sugiera un efecto sinérgico de estas sustancias sobre la hematopoyesis, no puede descartarse que, en conjunto, actúen como factores desencadenantes en individuos genéticamente predispuestos. La ausencia de infecciones agudas y de otras causas identificables que expliquen la trombocitopenia severa refuerza la necesidad de considerar estas sustancias dentro del diagnóstico diferencial, especialmente en contextos donde son frecuentes la automedicación o la exposición ambiental a plaguicidas.

El caso presentado pone de manifiesto la importancia de un enfoque multidisciplinario en el abordaje de enfermedades hematológicas severas de etiología incierta, así como la importancia de una historia clínica exhaustiva. En particular, subraya la necesidad de considerar factores farmacológicos y tóxicos como posibles causas de plaquetopenia severa, especialmente en pacientes con antecedentes de automedicación o exposición ambientales u ocupacionales, como ocurrió en nuestro paciente. Si bien los corticoides son eficaces en el tratamiento de la PTA, persiste la duda razonable de si la recuperación del recuento plaquetario fue atribuible exclusivamente a la suspensión de los agentes involucrados, o si los corticoides desempeñaron un rol decisivo al inducir la reversibilidad del cuadro.

En este sentido, esta comunicación pretende llamar la atención sobre la posible participación de sustancias poco estudiadas en la génesis de plaquetopenia severa y estimular la documentación sistemática de casos similares. Finalmente, la preocupante disponibilidad de fármacos como el orlistat sin prescripción médica, así como el uso indiscriminado de plaguicidas sin medidas de protección adecuadas, deben llamar la atención de las autoridades sanitarias y de los organismos reguladores, con el fin de prevenir eventos adversos de origen evitable.
